# Protective Effects of Phoenixin‐14 Administration Against Renal Ischemia/Reperfusion Injury in Rats

**DOI:** 10.1002/jbt.70200

**Published:** 2025-03-02

**Authors:** Samet Öz, Mehmet Refik Bahar, Güldeniz Şekerci, Aslı Taşlıdere, Suat Tekin

**Affiliations:** ^1^ Department of Veterinary Medicine Osmaniye Korkut Ata University, Vocational School of Health Services Osmaniye Turkey; ^2^ Osmaniye Korkut Ata University Faculty of Health Sciences Osmaniye Turkey; ^3^ Department of Physiology Inonu University, Faculty of Medicine Malatya Turkey; ^4^ Department of Histology and Embryology Inonu University, Faculty of Medicine Malatya Turkey

**Keywords:** Acute Kidney Injury, Antioxidant Activity, Oxidative Stress, Phoenixin‐14

## Abstract

Phoenixin (PNX), identified in the rat hypothalamus in 2013, has two bioactive isoforms with 14 and 20 amino acids. Initially studied for its role in reproductive regulation, research has since shown that PNX also prevents visceral pain, enhances memory, and aids heart tissue recovery. However, its role in kidney tissue remains unclear. Due to its antioxidant properties, PNX may help reduce oxidative stress and cellular damage in organs. This study was designed to determine the potential protective effects of Phoenixin‐14 (PNX‐14) against renal ischemia/reperfusion (I/R)‐induced injury in rats. 40 male *Wistar Albino* rats were divided into four groups: Control, I/R, PNX‐14 (50 µg/kg), and PNX‐14 (100 µg/kg) (*n* = 10). All groups except the control group underwent 45 min of bilateral ischemia followed by 24 h of reperfusion. PNX‐14 (50 and 100 μg/kg, intraperitoneally) was administered 1 h before induction of ischemia. Both doses of PNX‐14 reduced the levels of acute kidney injury markers (blood urea nitrogen and creatinine) in blood tissue (*p* < 0.05). PNX‐14 increased the activity of antioxidant enzymes (superoxide dismutase and catalase) and the levels of glutathione, while reducing malondialdehyde (*p* < 0.05). Histological evaluation of the I/R group revealed significant histopathological findings, and it was found that PNX‐14 administration improved these histological damages (*p* < 0.05). These results suggest that PNX‐14 provides protection against renal injury induced by I/R. After further studies, PNX‐14 may be a new therapeutic strategy to prevent renal I/R injury.

## Graphical abstract

Based on our biochemical and histopathological findings, we suggest that Phoexinin‐14, a newly discovered peptide, plays a protective role by activating antioxidant systems against acute kidney injury caused by ischemia/reperfusion injury.

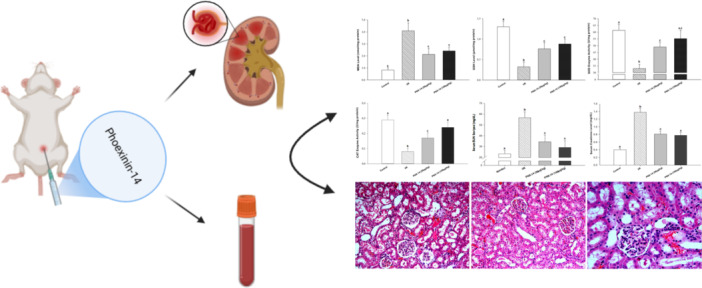

## 1 Introduction

Acute kidney injury (AKI) is characterized by accumulation of nitrogenous metabolic wastes, failure to maintain fluid‐electrolyte and acid–base balance, and a rapid decline in glomerular filtration rate (GFR) [1]. AKI is a clinical condition with high mortality and morbidity globally, affecting a large population. In addition to high mortality and morbidity, it also has deleterious effects that can lead to the onset of chronic kidney disease or the emergence of cardiovascular disorders. There are multiple risk factors for AKI, and the underlying pathophysiological mechanisms are complex [2, 3]. However, most researchers agree that ischemic acute tubular injury leads to loss of renal autoregulation, resulting in increased levels of vasoconstrictors that cause hypoperfusion and ischemia/reperfusion (I/R) injury and subsequent AKI [4]. Long‐term studies with various criteria for the diagnosis of AKI reveal a significant increase in incidence. The increase in the elderly population and the prevalence of chronic diseases are important reasons for the increase in the diagnosis of AKI [5]. The prevalence of the disease is increasing globally, with around 13.3 million cases and 1.7 million deaths each year [6].

Tissue I/R injury occurs when the blood supply to a given organ is suddenly and temporarily interrupted. In this context, I/R is typically associated with inflammation, oxidative stress and cellular apoptosis resulting from hypoxia and reperfusion [7]. During the ischemic period, reduced blood flow to the tissue leads to a decrease in ATP levels, resulting in decreased oxygenation and the onset of mitochondrial dysfunction that impairs cellular metabolic activities [8]. In the absence of oxygen, cellular aerobic metabolism is impaired and anaerobic metabolism becomes dominant. The accumulation of lactate due to anaerobic ATP production, combined with the inability to eliminate other metabolic wastes, leads to cellular acidosis and ultimately to a complete cessation of energy production [9]. The reperfusion phase after ischemia occurs with the generation of reactive oxygen species (ROS) and the migration and activation of leukocytes to the affected area, resulting in a cascade that ultimately exacerbates tissue damage [10]. ROS serve a protective function against diseases such as cancer, fight infections and facilitate phagocytosis, but excessive ROS production can damage lipids, proteins and deoxyribonucleic acid due to the formation of malondialdehyde (MDA) within the cell [11, 12]. Enzymatic antioxidant defense mechanisms such as glutathione (GSH), superoxide dismutase (SOD) and catalase (CAT) reduce oxidative stress by neutralizing radical metabolites, preventing their formation, repairing cellular damage, stopping radical‐producing chain reactions or increasing endogenous antioxidant capacity [13]. Therapies aimed at inhibiting or neutralizing ROS formation have been widely investigated in the prevention and treatment of AKI, which has recently become increasingly common.

Phoenixin (PNX) is a pleiotropic neuropeptide that was first discovered in the rat hypothalamus by Yosten et al. in 2013 and plays a role in the control of hormone release by the pituitary gland. PNX is composed of peptides containing 14, 17, 20 and 36 amino acids, but the bioactive forms are Phoenixin‐14 (PNX‐14) and Phoenixin‐20 (PNX‐20) [14, 15]. The amino acid sequence of PNX‐14 is identical in humans, cattle, pigs, mice, rats and chickens, while the amino acid sequence of PNX‐20 differs in one amino acid in humans and rodents [15, 16]. In addition to the brain, PNX has been found in peripheral tissues such as the heart, thymus, esophagus, stomach, spleen, pancreas, lung and kidney. Initial studies of the peptide showed that PNX‐14 primarily regulates reproductive activities; however, subsequent studies have documented that the peptide also plays a role in insulin activity, anxiety, pain perception, memory and food consumption [15]. In addition to psychological and physiological stress, research confirms the preventive effects of PNX on oxidative stress and inflammatory pathways [17]. The antioxidant properties of PNX clearly indicate that the peptide may also protect and suppress inflammatory processes in other organs and may be positioned as a promising therapeutic agent for certain disorders. Based on this, we hypothesize that PNX‐14, due to its antioxidant properties and effects on inflammatory processes, may play a protective role by reducing oxidative stress and cellular damage caused by I/R in kidney tissue. In light of these data, the aim of our study was to investigate the possible effects of PNX‐14 on renal I/R‐induced cell death and oxidative stress in rats.

## 2 Material and Methods

### 2.1 Animals

Ethical approval for all experimental procedures and testing protocols involving animals was obtained from the Ethics Committee of Animal Experiments, Inonu University Faculty of Medicine (Approval No: 2024/14‐2). The study used 40 male *Wistar Albino* rats, each weighing between 250 and 300 g. The animals were individually housed in stainless steel cages under controlled temperature (22 ± 1°C) and a 12 h light/dark cycle. They had unlimited access to commercial standard chow and drinking water.

### 2.2 Experimental Design

**Figure 1:**
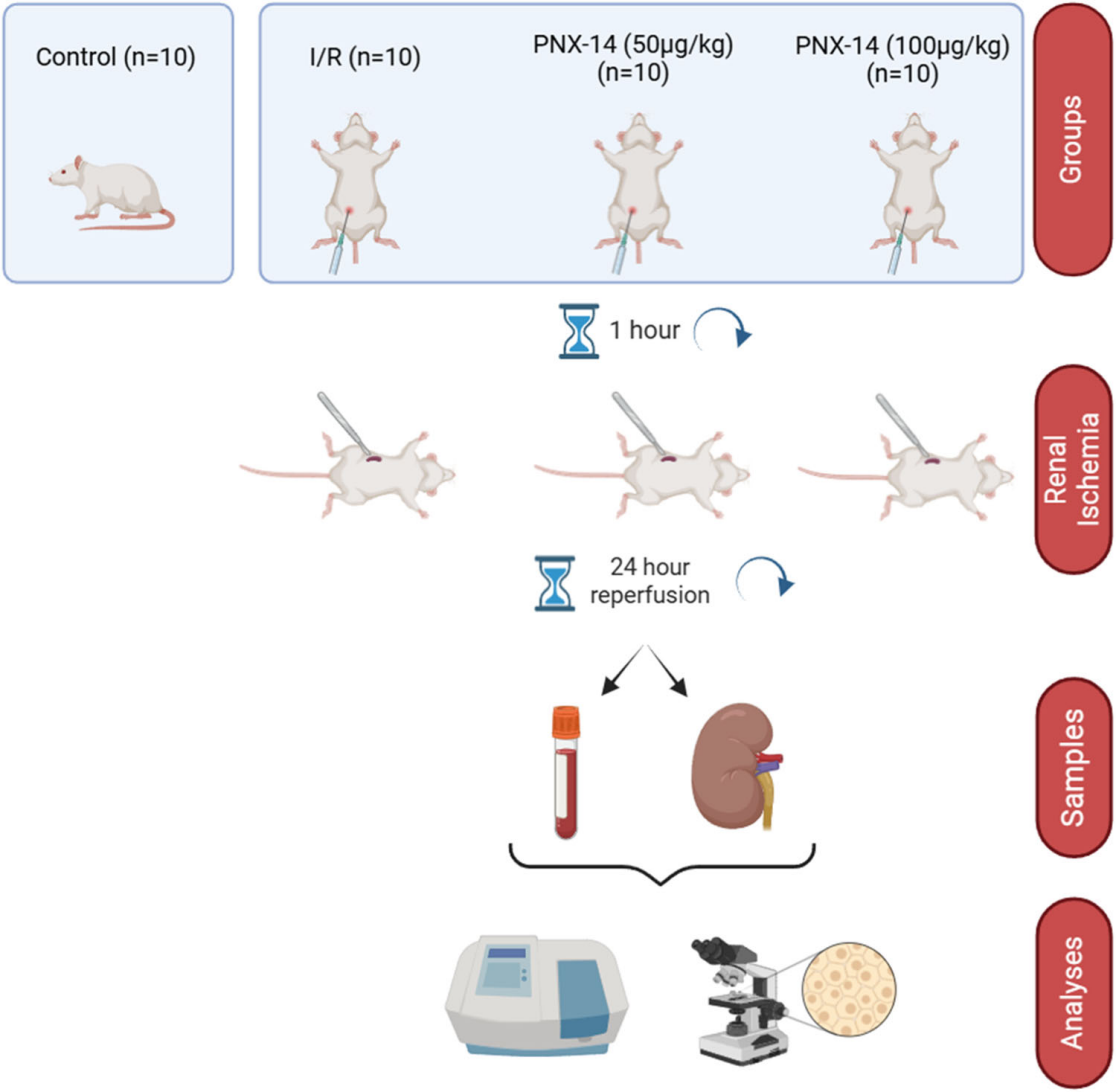
Schematic representation of the experimental design of the study.

Animals were randomly assigned to groups based on their body weights using a computer algorithm and divided into 4 groups (Control, I/R, PNX‐14 (50 µg/kg), and PNX‐14 (100 µg/kg)) with 10 animals per group (*n* = 10). Before surgery, all animals were anesthetized intraperitoneally (ip) with 70 mg/kg ketamine (Richter Pharma AG, Australia) and 8 mg/kg xylazine (Alfazyn, Netherlands). The surgical area was shaved and disinfected with povidone‐iodine solution. No surgical procedure was performed on the animals in the Control group. In the I/R group, serum physiological solution (the solvent for PNX‐14) was administered ip 1 h before ischemia. In the treatment groups, two different doses of PNX‐14 (Lot Number: MTFA30931‐0118, NovoPro), 50 and 100 µg/kg [18], were administered ip 1 h before [19] ischemia. Subsequently, animals in the I/R and treatment groups underwent 45 min of ischemia followed by 24 h of reperfusion in both kidneys [20, 21]. After reperfusion, the animals were decapitated, and blood and kidney tissue samples were collected. Blood samples were centrifuged. Serum and kidney tissue samples for biochemical analysis were stored in a deep freezer at −80°C, while kidney tissue samples for histological analysis were preserved in a solution containing 10% formalin. Experimental flow diagram is given in Figure 1.

### 2.3 Biochemical Analyses

#### 2.3.1 Blood Urea Nitrogen and Creatinine

Blood urea nitrogen (BUN) and creatinine serum levels were analyzed using an Olympus Autoanalyzer (Olympus Instruments, Tokyo, Japan).

#### 2.3.2 Malondialdehyde

MDA concentrations in tissues were analyzed using the Mihara and Uchiyama technique [22]. 0.5 ml of tissue homogenate was combined with 3 ml of 1% H_3_PO_4_ and 1 ml of 0.6% thiobarbituric acid solution and incubated at 90°C for 45 min. The mixture was allowed to cool to room temperature, then 4 ml of n‐butanol was added and shaken. The combination was centrifuged, and the resulting n‐butanol phase was read spectrophotometrically at 535 nm. MDA concentrations were determined using 1,1,3,3,3‐Tetraethoxypropane standard curve. MDA concentrations of the samples were measured as nmol/g tissue.

#### 2.3.3 Glutathione

GSH concentrations in kidney tissue samples were analyzed using the Ellman technique [23]. Equal volumes of 10% trichloroacetic acid solution were added to the homogenates and then centrifuged. A solution of 5,5′‐Dithiobis‐2‐nitrobenzoic acid (DTNB) in 1% sodium citrate was read at 410 nm using a spectrophotometer (Thermo, USA). Calculations were performed based on standard graphs of the determined GSH values.

#### 2.3.4 Superoxide Dismutase Enzyme Activity

The activity of SOD enzyme in the samples was analyzed using the technique of Sun et al. [24]. The aim is to inhibit the reduction of nitro blue tetrazolium (NBT) by the xanthine‐xanthine oxidase system, which acts as a superoxide generator. One SOD unit is defined as the amount of enzyme that causes 50% inhibition of the NBT reduction rate. SOD activity was calculated as U/g protein.

#### 2.3.5 Catalase Enzyme Activity

CAT enzyme activity was analyzed using the Aebi technique [25]. Absorbance measurements were performed using a H_2_O_2_ solution produced with pH 7.0 and 50 mM phosphate buffer. An initial absorbance adjustment of 0.5 nm was made for the H_2_O_2_ solution. Phosphate buffer was used as a control. The H_2_O_2_ solution was diluted with 25 ml of phosphate buffer, which served as substrate. First, 2 µL of supernatant and then 10 µL of H_2_O_2_ were added to the wells. The absorbance decrease was recorded every 15 s for a period of 5 min. Measurements were performed at a wavelength of 240 nm.

#### 2.3.6 Protein Concentration

The Lowry method was used for protein assay [26]. Bovine serum albumin was used as a standard, and a calibration curve was constructed. Analyses were then performed according to the protocol. Results were presented in micrograms per milliliter (µg/mL).

### 2.4 Kidney Histopathology

Kidney samples were preserved in 10% formalin for histological evaluation. The samples underwent standard tissue processing procedures and were embedded in paraffin. The paraffin‐embedded samples were sectioned at a thickness of 5 µm, mounted on slides, and then stained with Hematoxylin‐Eosin (HE). Tissue samples were analyzed using a light microscope (Leica DFC280) and a Leica Q Win Image Analysis System (Leica Micros Imaging Solutions Ltd., Cambridge, UK).

The histopathological analysis of tissue damage was based on various parameters, including tubular lumen dilation, hemorrhage, inflammatory cell infiltration, hydropic degeneration, hemorrhage between tubules and glomeruli, epithelial atrophy, cell desquamation in tubules, vacuolization of tubular epithelial cells, and the presence of debris in the tubular lumen. The samples were analyzed semi‐quantitatively by examining at least five microscopic fields per slide. Each sample was evaluated using a scale from 0 to 3 for each criterion (0: none, 1: mild, 2: moderate, 3: severe). The total scores were calculated based on these characteristics [27]. Microscopic evaluation and cell counting were performed blindly by the same histologist.

### 2.5 Statistical Analyses

Analyses were performed using IBM SPSS Statistics 22.0 software for Windows. Quantitative data were summarized using mean ± standard deviation (SD). The Shapiro‐Wilk test was used to assess the normality of distribution. The Kruskal‐Wallis H test was used to compare quantitative variables between groups, and when a significant difference was detected, pairwise comparisons were performed using the Bonferroni‐corrected Mann‐Whitney U test. *p* < 0.05 was considered statistically significant.

## 3 Results

### 3.1 Serum Blood Urea Nitrogen and Creatinine Levels

**Figure 2:**
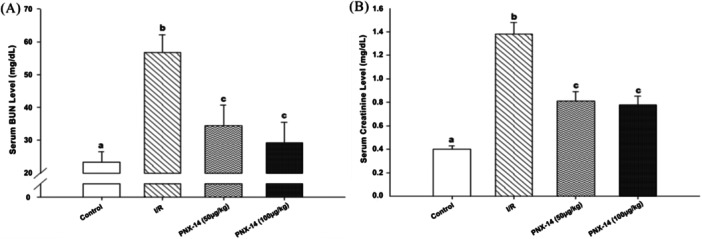
Effect of PNX‐14 on serum (A) BUN and (B) creatinine levels in renal I/R injury. Values are expressed as mean ± SD (^a,b,c^ *p* < 0.05). (I/R: ischemia/reperfusion group, PNX‐14 (50 µg/kg): Phoenixin‐14 50 µg/kg, PNX‐14 (100 µg/kg): Phoenixin‐14 100 µg/kg, BUN: blood urea nitrogen).

The serum BUN and creatinine levels of the groups are shown in Figure 2(A, B). In the I/R group, there was a significant increase in serum BUN and creatinine levels compared to the control group (*p* < 0.05). In the PNX‐14 treated groups (50 and 100 µg/kg), however, there was a significant decrease in serum BUN and creatinine levels (*p* < 0.05).

### 3.2 Biochemistry Results

**Figure 3:**
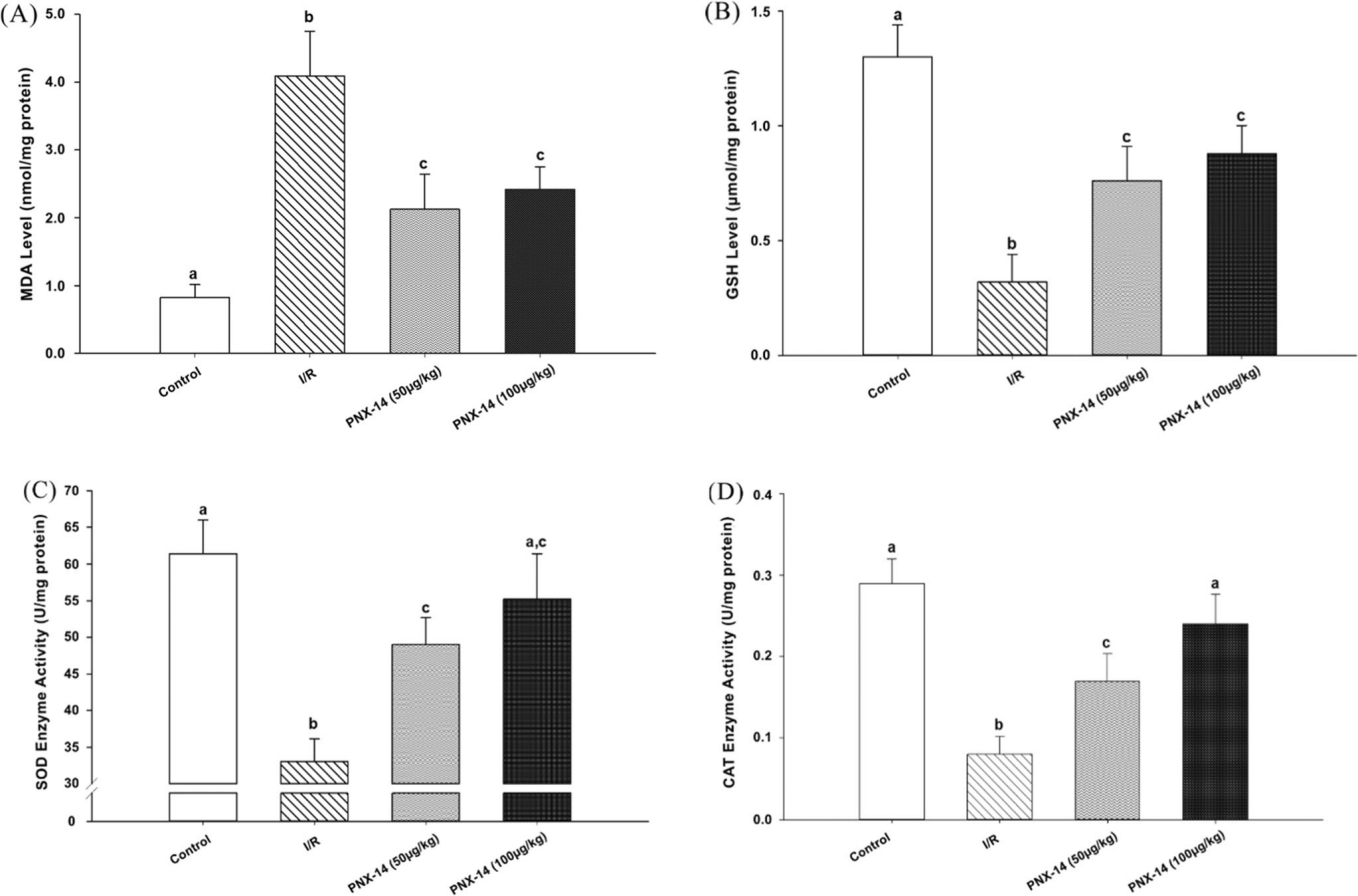
(A) MDA, (B) GSH, (C) SOD and (D) CAT levels in kidney tissue. In general, antioxidant enzyme levels were decreased in the I/R group (B–D). Furthermore, lipid peroxidation (MDA level) was increased in kidney tissue following ischemia (A). However, PNX‐14 administration ameliorated ischemia‐induced oxidative damage. Values are expressed as mean ± SD (^a,b,c^ *p* < 0.05). (I/R: ischemia/reperfusion group, PNX‐14 (50 µg/kg): Phoenixin‐14 50 µg/kg, PNX‐14 (100 µg/kg): Phoenixin‐14 100 µg/kg. MDA: Malondialdehyde. GSH: Glutathione; SOD: Superoxide dismutase; CAT: Catalase).

Biochemistry results for all groups are presented as follows: MDA level in kidney tissue was increased in the I/R group compared to the other groups (*p* < 0.05). However, MDA levels decreased in PNX‐14 treated animals compared to the I/R group (Figure 3(A); *p* < 0.05). Figure 3 GSH, SOD and CAT levels in kidney tissue were significantly increased in PNX‐14 treated groups compared to I/R group (Figure 3(B‐C‐D); *p* < 0.05). In addition, SOD and CAT levels in PNX‐14 treated animals were similar to those in the control group (Figure 3(C‐D); *p* > 0.05).

### 3.3 Histopathologic Findings

**Figure 4:**
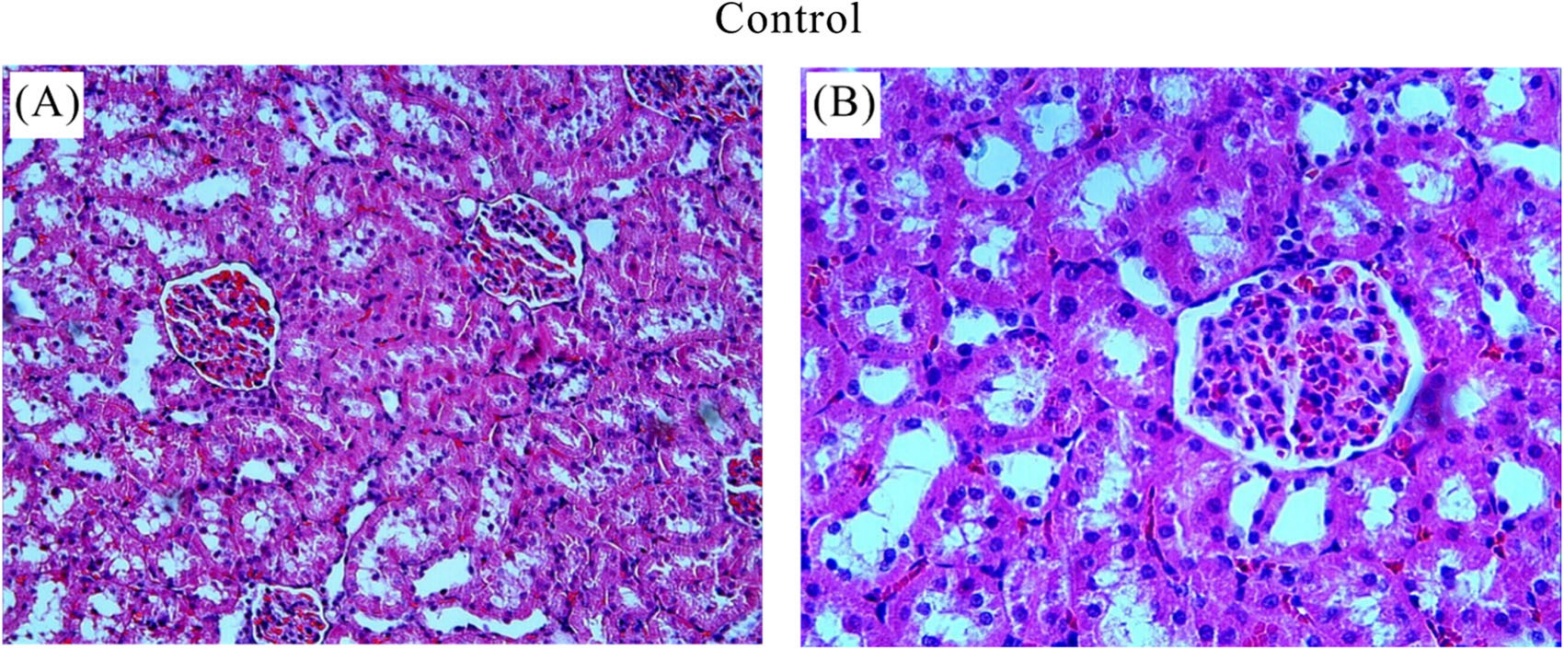
Histologic images of the control group. Kidney tissue from the control animal showed normal tubules as well as normal sized glomeruli (A: HE x20; B: HE x40).

**Figure 5:**
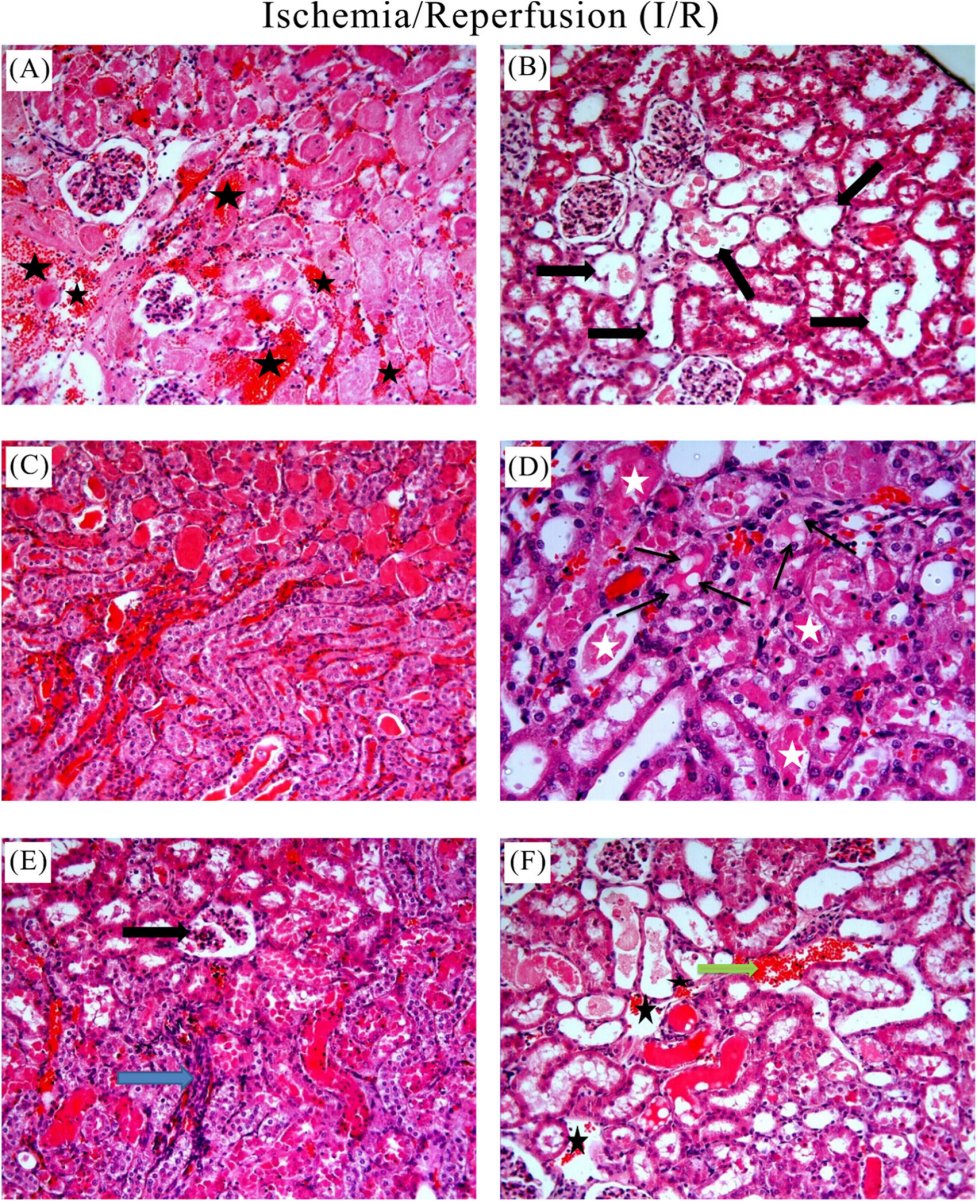
Histologic images of the I/R group. Degeneration and hemorrhage in tubule and glomerulus structures (A; black star), dilatation in tubules (B; black arrows), shedding of tubule epithelial cells, vacuolization in tubules (D; black arrows), fluid accumulation in tubule lumen (D; white stars), degeneration of glomerular structures (E; black arrow), mononuclear cell infiltration (E; blue arrow), fluid accumulation and vascular congestion in the tubule lumen (F; green arrow), hemorrhage (F; black star) (A, B, C, E, F: HE x20; D: HE x40).

**Figure 6:**
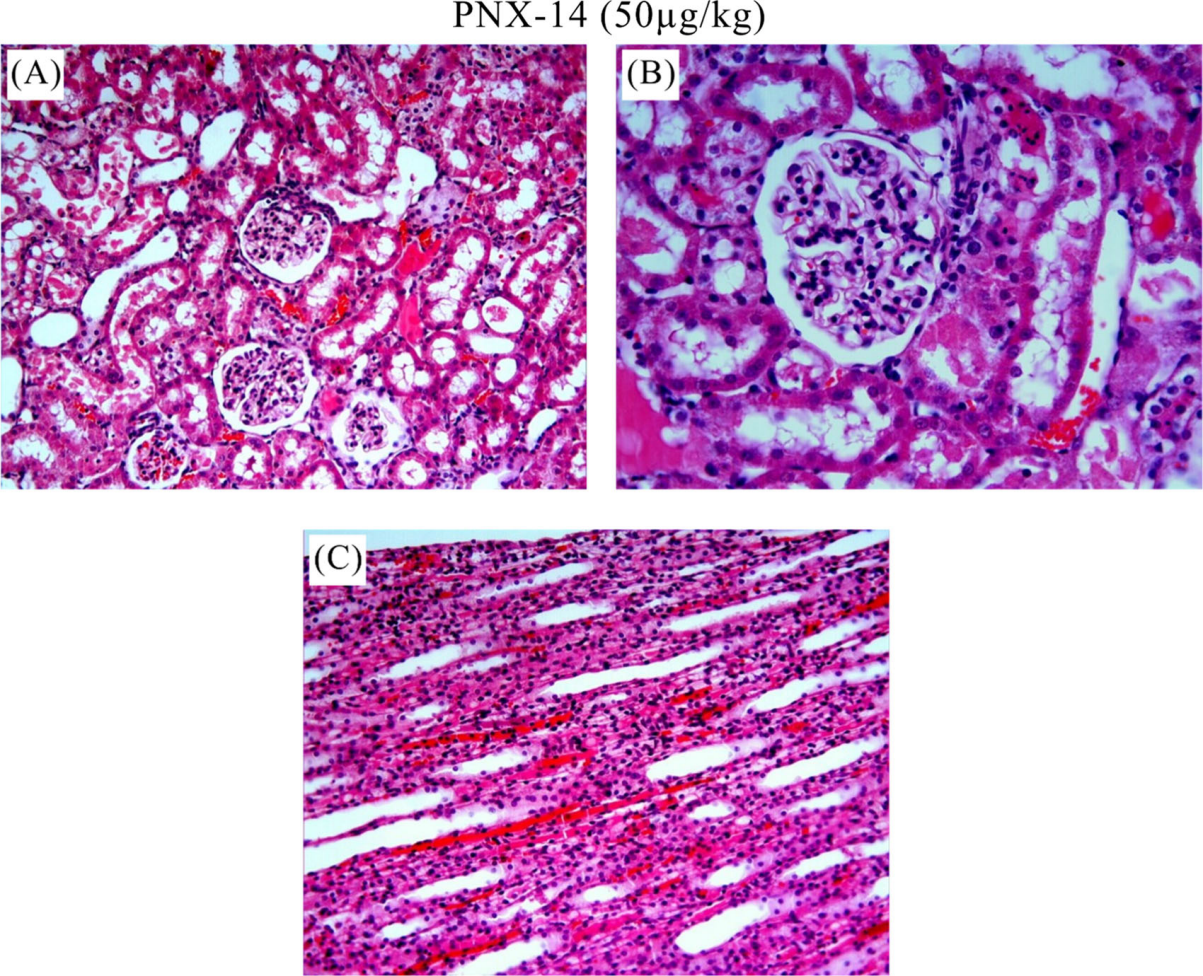
Histologic images of PNX‐14 (50 µg/kg) group. Small amount of tubule epithelial cell shedding, small amount of fluid accumulation in tubule lumen, small amount of tubule lumen enlargement (A), small amount of tubule epithelial cell shedding, small amount of fluid accumulation in tubule lumen (B), small amount of hemorrhage between tubules compared to I/R group (C). A, C: HE x20; B: HE x40).

**Figure 7:**
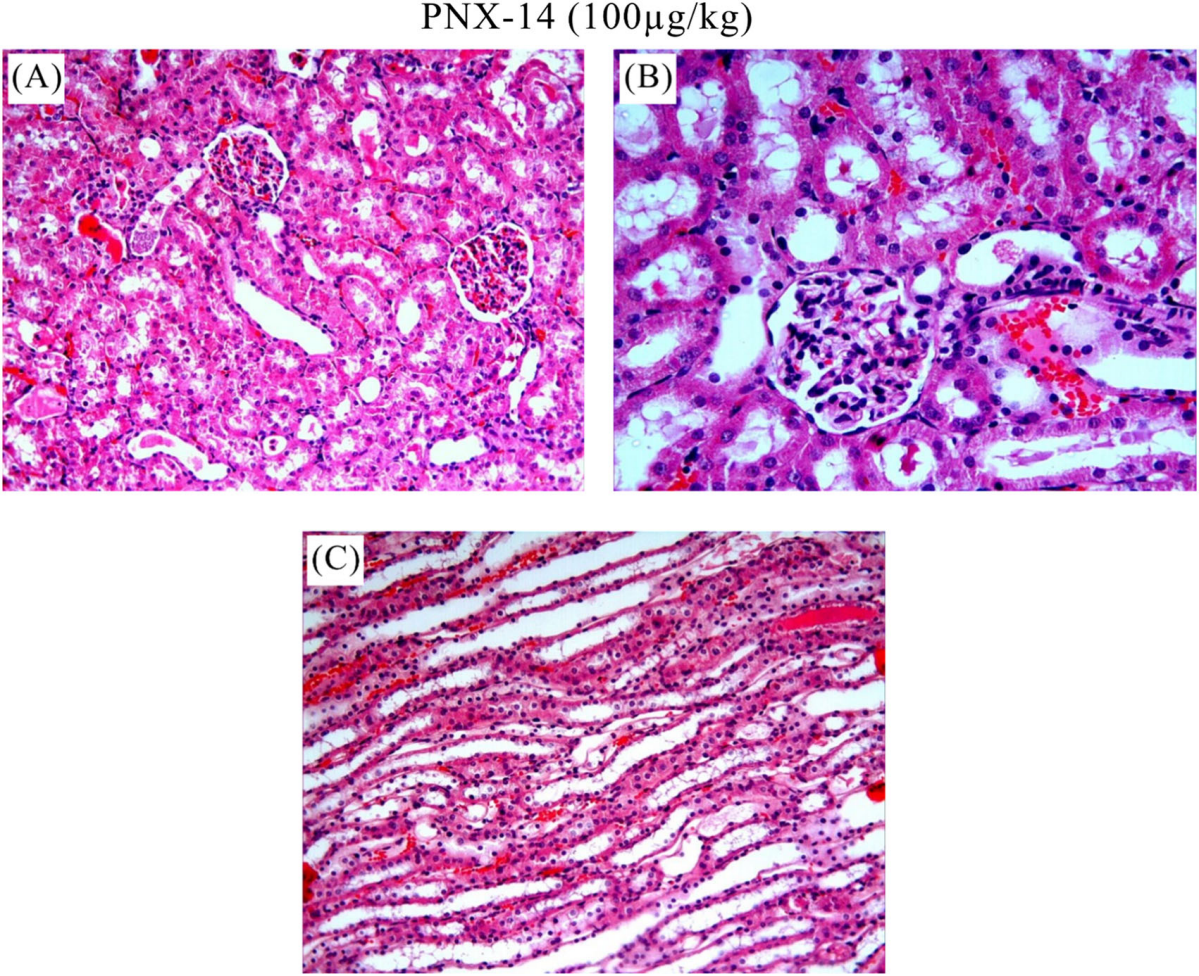
Histological images of PNX‐14 (100 µg/kg) group, significant decrease in all findings compared to I/R group (A), very little edema and very little hemorrhage (B, C). A: HE x20; B, C: HE x40.

The control group showed normal histologic appearance (Figure 4(A, B)). In the I/R group, heterogeneous changes and significant pathologic damages were observed in the kidney sections (Figure 5). Degeneration and hemorrhage in tubules and glomerular structures (Figure 5(A)), dilatation in tubules (Figure 5(B)), fluid accumulation and hemorrhage in tubule lumen (Figure 5(C), shedding of tubule epithelial cells, vacuolization in tubules, fluid accumulation and hemorrhage in tubule lumen (Figure 5(D), degeneration of glomerular structures, mononuclear cell infiltration, shedding of tubule epithelial cells, fluid accumulation and hemorrhage in the tubule lumen (Figure 5(E), fluid accumulation and vascular congestion in the tubule lumen (Figure 5(F)) were histopathological lesions observed in the I/R group. All treatment groups showed reduced renal injury compared to the I/R group. As shown in Figure 6 and Figure 7, we observed improvement in the histologic appearance of renal tubules and glomeruli in the treatment groups. Renal scores of the groups are presented in Table 1.

**Table 1:** Histologic scores of the groups.

Groups	Histopathologic kidney damage (Mean ± SD)
Control	0.58 ± 0.07^a^
I/R	2.27 ± 0.09^b^
PNX‐14 (50 µg/kg)	1.70 ± 0.09^c^
PNX‐14 (100 µg/kg)	1.33 ± 0.10^c^

*Note:* Mean differences between values with different superscript letters in the same column are statistically significant (^a,b,c^ *p* ≤ 0.0001).

## 4 Discussion

AKI can be defined as a decrease in GFR over a period of time ranging from hours to days [28]. Decreased GFR leads to accumulation of nitrogenous wastes in the bloodstream, especially serum BUN and creatinine [29]. Under normal conditions, the kidneys respond to reduced blood flow by autoregulatory mechanisms and can regulate GFR accordingly. However, in severe ischemic conditions, the supply of oxygen and metabolic substrates to the kidneys becomes inadequate [30]. During I/R injury, renal tissue produces excess ROS that exceeds the body's elimination capacity. ROS interact with cellular structural components, causing oxidative cellular damage [31]. This disrupts the structural and functional integrity of the cell membrane through lipid peroxidation in mitochondria, lysosomes and plasma membranes. MDA and thiobarbituric acid reactive compounds resulting from lipid peroxidation are defined as the most important biomarkers of oxidative stress [32]. CAT, SOD, and GSH are endogenous enzymes that metabolize free radicals and are required to scavenge ROS [33].

PNX‐14, whose effect against renal I/R injury was investigated in our study, was first identified in rat hypothalamus in 2013. Initially thought to be a regulator of the reproductive system [15], ongoing studies on PNX‐14 have reported that the peptide has significant anti‐inflammatory and antioxidant effects [34]. In fact, in a study conducted by Yılmaz et al., it was emphasized that PNX‐14 decreased MDA level and increased SOD enzyme activity in the I/R injury model created in testicular tissue [35]. In another study investigating the relationship between PNX‐14 and oxidative damage, it was reported that PNX‐14 showed an antioxidative stress effect against LPS‐induced damage in astrocytes by suppressing lipopolysaccharide (LPS)‐induced excessive ROS production and decrease in SOD level [17]. In another study, PNX‐20, another isoform of PNX, was observed to reduce MDA levels and suppress oxidative stress by increasing SOD enzyme activity in rats modeled with pulmonary arterial hypertension [36]. In a study in which nonalcoholic fatty liver disease model was established in rats, it was reported that PNX‐14 decreased MDA level, increased SOD and GSH enzyme levels and reduced oxidative stress as a result [19]. Zandeh‐Rahimi et al. emphasized that PNX‐14 showed significant antioxidant effects against indomethacin‐induced duodenal ulcer by increasing CAT and SOD levels [18].

In our study, serum BUN and creatinine levels of PNX‐14 treated rats were found to be lower compared to the I/R group. We also observed that GSH, SOD and CAT levels in the kidney tissues of PNX‐14‐treated animals increased compared to the I/R group, while MDA level, an important indicator of oxidative stress, decreased compared to the I/R group. The data we obtained indicate that PNX‐14 may be effective in preventing AKI in the I/R process. These results were consistent with the results of previous experimental studies with PNX‐14.

Studies have shown that renal I/R reduces renal blood flow as a result of reduced renal vascular sensitivity and microvascular damage. This leads to inflammatory cell infiltration, glomerular damage, tubular epithelial cell destruction, apoptosis and fibrosis [37]. Previous studies in the literature have shown that PNX‐14 significantly reduces intracranial intima damage and inflammatory cell infiltration by histopathological analysis [38]. In our study, heterogeneous changes and significant pathological damages were observed in the kidney sections in the I/R group. Degeneration and hemorrhage in tubules and glomerular structures, vacuolization and dilatation in tubules, vascular congestion, fluid accumulation and hemorrhage in tubule lumen, shedding of tubule epithelial cells, degeneration in glomerular structures and mononuclear cell infiltration were detected. In the PNX‐14‐treated groups, renal damage was significantly reduced compared to the I/R group. The results of the present study were in support of the existing studies in the literature. Based on our biochemical and histopathological findings, we can conclude that PNX‐14, a newly discovered peptide, plays a protective role by activating antioxidant systems against AKI induced by I/R injury.

## 5 Conclusion

In conclusion, biochemical and histological analyses revealed that PNX‐14 played a protective role in renal I/R injury experimentally induced in rats. Although these protective effects of PNX‐14 on renal tissue are not reported in the existing literature, our findings are the first in this field. Although experimental studies have been conducted with many promising pharmacologic agents to prevent I/R injury, there is a need to investigate the effects of these new protective peptides on other tissues and organs to find a clinical application.

## Author Contributions

**Samet Özamet Özamet Öz:** investigation, methodology, data curation, validation, writing–original draft. **Mehmet Refik Bahar:** data curation, investigation. **Güldeniz Şekerciüldeniz Şekerciüldeniz Şekerci:** investigation, formal analysis. **Aslı Taşlıdere:** data curation,methodology. **Samet Özamet Özuat Tekin:** writing–review and editing, conceptualization, Samet Özamet Özupervision.

## Ethics Statement

Ethical approval for all experimental procedures and testing protocols involving animals was obtained from the Ethics Committee of Animal Experiments, Inonu University Faculty of Medicine (Approval No: 2024/14‐2).

## Conflicts of Interest

The authors declare no conflicts of interest.

## Data Availability

The data that support the findings of this study are available from the corresponding author upon reasonable request.

## References

[1] J. Wang , D. Wang , Y. Li , et al., “Rhabdomyolysis‐Induced Acute Kidney Injury Under Hypoxia and Deprivation of Food and Water,” Kidney and Blood Pressure Research 37, no. 4–5 (2013): 414–421. 24247301 10.1159/000350154

[2] K. Hahn , M. Kanbay , M. A. Lanaspa , R. J. Johnson , and A. A. Ejaz , “Serum Uric Acid and Acute Kidney Injury: A Mini Review,” Journal of Advanced Research 8, no. 5 (2017): 529–536. 28748118 10.1016/j.jare.2016.09.006 PMC5512150

[3] E. V. Schrezenmeier , J. Barasch , K. Budde , T. Westhoff , and K. M. Schmidt‐Ott , “Biomarkers in Acute Kidney Injury–Pathophysiological Basis and Clinical Performance,” Acta Physiologica 219, no. 3 (2017): 556–574. 10.1111/apha.12764 PMC5575831 27474473

[4] P. Devarajan , “Update on Mechanisms of Ischemic Acute Kidney Injury,” Journal of the American Society of Nephrology 17, no. 6 (2006): 1503–1520. 16707563 10.1681/ASN.2006010017

[5] M. G. Mercado , D. K. Smith , and E. L. Guard , “Acute Kidney Injury: Diagnosis and Management,” American Family Physician 100, no. 11 (2019): 687–694. 31790176

[6] P. Pickkers , M. Darmon , E. Hoste , et al., “Acute Kidney Injury in the Critically Ill: An Updated Review on Pathophysiology and Management,” Intensive Care Medicine 47, no. 8 (2021): 835–850. 34213593 10.1007/s00134-021-06454-7 PMC8249842

[7] M. Malek and M. Nematbakhsh , “Renal Ischemia/Reperfusion Injury; From Pathophysiology to Treatment,” Journal of Renal Injury Prevention 4, no. 2 (2015): 20–27. 26060833 10.12861/jrip.2015.06 PMC4459724

[8] N. Pabla and A. Bajwa , “Role of Mitochondrial Therapy for Ischemic‐Reperfusion Injury and Acute Kidney Injury,” Nephron 146, no. 3 (2022): 253–258. 34883481 10.1159/000520698 PMC9090938

[9] M. Kosieradzki and W. Rowiński , “Ischemia/Reperfusion Injury in Kidney Transplantation: Mechanisms and Prevention,” Transplantation Proceedings 40, no. 10 (2008): 3279–3288. 19100373 10.1016/j.transproceed.2008.10.004

[10] H. K. Eltzschig and T. Eckle , “Ischemia and Reperfusion—From Mechanism to Translation,” Nature Medicine 17, no. 11 (2011): 1391–1401. 10.1038/nm.2507 PMC3886192 22064429

[11] N. Petejova and A. Martinek , “Acute Kidney Injury Due to Rhabdomyolysis and Renal Replacement Therapy: A Critical Review,” Critical Care 18, no. 3 (2014): 224. 25043142 10.1186/cc13897 PMC4056317

[12] S. Sen and R. Chakraborty , The Role of Antioxidants in Human Health. Oxidative Stress: Diagnostics, Prevention, and Therapy (ACS Publications, 2011), 1–37.

[13] H. Karabulut and M. Ş. Gülay , “Antioksidanlar,” Veterinary Journal of Mehmet Akif Ersoy University 1, no. 1 (2016): 65–76.

[14] B. Zhang and J. Li , “Phoenixin‐14 Protects Human Brain Vascular Endothelial Cells Against Oxygen‐Glucose Deprivation/Reoxygenation (OGD/R)‐Induced Inflammation and Permeability,” Archives of Biochemistry and Biophysics 682 (2020): 108275. 31962109 10.1016/j.abb.2020.108275

[15] G. L. C. Yosten , R. M. Lyu , A. J. W. Hsueh , et al., “A Novel Reproductive Peptide, Phoenixin,” Journal of Neuroendocrinology 25, no. 2 (2013): 206–215. 22963497 10.1111/j.1365-2826.2012.02381.x PMC3556183

[16] A. Cowan , R.‐M. Lyu , Y.‐H. Chen , S. L. Dun , J.‐K. Chang , and N. J. Dun , “Phoenixin: A Candidate Pruritogen in the Mouse,” Neuroscience 310 (2015): 541–548. 26415767 10.1016/j.neuroscience.2015.09.055 PMC4633352

[17] J. Wang , B. Zheng , S. Yang , X. Tang , J. Wang , and D. Wei , “The Protective Effects of Phoenixin‐14 Against Lipopolysaccharide‐Induced Inflammation and Inflammasome Activation in Astrocytes,” Inflammation Research 69, no. 8 (2020): 779–787. 32435966 10.1007/s00011-020-01355-9

[18] Y. Zandeh‐Rahimi , N. Panahi , S. Hesaraki , and S. H. Shirazi‐Beheshtiha , “Protective Effects of Phoenixin‐14 Peptide in the Indomethacin‐Induced Duodenal Ulcer: An Experimental Study,” International Journal of Peptide Research and Therapeutics 28, no. 1 (2022): 43. 35002587 10.1007/s10989-021-10314-9 PMC8722415

[19] F. Yang , P. Huang , L. Shi , F. Liu , A. Tang , and S. Xu , “Phoenixin 14 Inhibits High‐Fat Diet‐Induced Non‐Alcoholic Fatty Liver Disease in Experimental Mice,” Drug Design, Development and Therapy 14 (2020): 3865–3874. 33061293 10.2147/DDDT.S258857 PMC7519838

[20] M. Çakır , S. Tekin , Z. Doğanyiğit , P. Çakan , and E. Kaymak , “The Protective Effect of Cannabinoid Type 2 Receptor Activation on Renal Ischemia–Reperfusion Injury,” Molecular and Cellular Biochemistry 462, no. 1 (2019): 123–132. 31446615 10.1007/s11010-019-03616-6

[21] S. Tekin , A. Beytur , M. Cakir , et al. “Protective Effect of Saxagliptin Against Renal Ischaemia Reperfusion Injury in Rats,” Archives of Physiology and Biochemistry 128, no. 3 (2020): 608–618, 10.1080/13813455.2020.1715442. 31979992

[22] M. Uchiyama and M. Mihara , “Determination of Malonaldehyde Precursor in Tissues by Thiobarbituric Acid Test,” Analytical Biochemistry 86, no. 1 (1978): 271–278. 655387 10.1016/0003-2697(78)90342-1

[23] G. L. Ellman , “Tissue Sulfhydryl Groups,” Archives of Biochemistry and Biophysics 82, no. 1 (1959): 70–77. 13650640 10.1016/0003-9861(59)90090-6

[24] Y. Sun , L. W. Oberley , and Y. Li , “A Simple Method for Clinical Assay of Superoxide Dismutase,” Clinical Chemistry 34, no. 3 (1988): 497–500. 3349599

[25] H. Aebi , “Catalase,” Methods of Enzymatic Analysis 2 (1974): 673–684.

[26] O. H. Lowry , N. J. Rosebrough , A. L. Farr , and R. J. J. Jb. C. Randall , “Protein Measurement With the Folin Phenol Reagent,” Journal of Biological Chemistry 193, no. 1 (1951): 265–275. 14907713

[27] E. Yilmaz , R. Melekoglu , O. Ciftci , S. Eraslan , A. Cetin , and N. Basak , “The Therapeutic Effects of Curcumin and Capsaicin Against Cyclophosphamide Side Effects on the Uterus in Rats,” Acta Cirurgica Brasileira 33 (2018): 499–507. 30020311 10.1590/s0102-865020180060000004

[28] G. Vivino , M. Antonelli , M. L. Moro , et al., “Risk Factors for Acute Renal Failure in Trauma Patients,” Intensive Care Medicine 24, no. 8 (1998): 808–814. 9757925 10.1007/s001340050670

[29] C. L. Edelstein , “Biomarkers of Acute Kidney Injury,” Advances in Chronic Kidney Disease 15, no. 3 (2008): 222–234. 18565474 10.1053/j.ackd.2008.04.003 PMC3287955

[30] C. Ronco , R. Bellomo , and J. A. Kellum , “Acute Kidney Injury,” Lancet 394, no. 10212 (2019): 1949–1964. 31777389 10.1016/S0140-6736(19)32563-2

[31] L. Huang , T. Belousova , M. Chen , G. DiMattia , D. Liu , and D. Sheikh‐Hamad , “Overexpression of Stanniocalcin‐1 Inhibits Reactive Oxygen Species and Renal Ischemia/Reperfusion Injury in Mice,” Kidney International 82, no. 8 (2012): 867–877. 22695329 10.1038/ki.2012.223 PMC3443530

[32] M. S. Paller , J. R. Hoidal , and T. F. Ferris , “Oxygen Free Radicals in Ischemic Acute Renal Failure in the Rat,” Journal of Clinical Investigation 74, no. 4 (1984): 1156–1164. 6434591 10.1172/JCI111524 PMC425281

[33] Y. Liu , L. Wang , Y. Du , et al., “Effects of Apigenin Pretreatment Against Renal Ischemia/Reperfusion Injury via Activation of the JAK2/STAT3 Pathway,” Biomedicine & pharmacotherapy = Biomedecine & Pharmacotherapie 95 (2017): 1799–1808. 28962085 10.1016/j.biopha.2017.09.091

[34] M. Billert , A. Rak , K. W. Nowak , and M. Skrzypski , “Phoenixin: More Than Reproductive Peptide,” International Journal of Molecular Sciences 21, no. 21 (2020): 8378. 33171667 10.3390/ijms21218378 PMC7664650

[35] N. Yilmaz , J. Hudaykulıyeva , and S. Gul , “Phoenixin‐14 May Ameliorate Testicular Damage Caused by Torsion‐Detorsion by Reducing Oxidative Stress and Inflammation in Prepubertal Rats,” Tissue and Cell 88 (2024): 102405. 38754242 10.1016/j.tice.2024.102405

[36] Y. Chai , X. Gu , H. Zhang , X. Xu , and L. Chen , “Phoenixin 20 Ameliorates Pulmonary Arterial Hypertension via Inhibiting Inflammation and Oxidative Stress,” Aging 16, no. 6 (2024): 5027–5037. 38517365 10.18632/aging.205468 PMC11006497

[37] S. K. Verma and B. A. Molitoris , “Renal Endothelial Injury and Microvascular Dysfunction in Acute Kidney Injury,” Seminars in Nephrology 35, no. 1 (2015): 96–107. 25795503 10.1016/j.semnephrol.2015.01.010 PMC4476528

[38] C. Ling , Y. Yang , B. Zhang , H. Wang , and C. Chen , “Phoenixin‐14 Maintains the Contractile Type of Vascular Smooth Muscle Cells in Cerebral Aneurysms Rats,” Journal of Biochemical and Molecular Toxicology 38, no. 9 (2024): e23813. 39148253 10.1002/jbt.23813

